# Urinalysis of individuals with renal hyperfiltration using ATR-FTIR spectroscopy

**DOI:** 10.1038/s41598-022-25535-1

**Published:** 2022-12-03

**Authors:** İlhan Kurultak, Neslihan Sarigul, Nil Su Kodal, Filiz Korkmaz

**Affiliations:** 1 Department of Nephrology, Faculty of Medicine, Trakya University, 22000 Edirne, Turkey; 2 Institute of Nuclear Science, Hacettepe University, Ankara, Turkey; 3 Department of Internal Medicine, Faculty of Medicine, Trakya University, Edirne, Turkey; 4 Biophysics Laboratory, Faculty of Engineering, Atilim University, Ankara, Turkey

**Keywords:** Molecular biophysics, Nephrology, Infrared spectroscopy

## Abstract

Abnormal increased glomerular filtration rate (GFR), otherwise known as renal hyperfiltration (RHf), is associated with an increased risk of chronic kidney disease and cardiovascular mortality. Although it is not considered as a disease alone in medicine today, early detection of RHf is essential to reducing risk in a timely manner. However, detecting RHf is a challenge since it does not have a practical biochemical marker that can be followed or quantified. In this study, we tested the ability of ATR-FTIR spectroscopy to distinguish 17 individuals with RHf (hyperfiltraters; RHf (+)), from 20 who have normal GFR (normofiltraters; RHf(−)), using urine samples. Spectra collected from hyperfiltraters were significantly different from the control group at positions 1621, 1390, 1346, 933 and 783/cm. Intensity changes at these positions could be followed directly from the absorbance spectra without the need for pre-processing. They were tentatively attributed to urea, citrate, creatinine, phosphate groups, and uric acid, respectively. Using principal component analysis (PCA), major peaks of the second derivative forms for the classification of two groups were determined. Peaks at 1540, 1492, 1390, 1200, 1000 and 840/cm were significantly different between the two groups. Statistical analysis showed that the spectra of normofiltraters are similar; however, those of hyperfiltraters show diversity at multiple positions that can be observed both from the absorbance spectra and the second derivative profiles. This observation implies that RHf can simultaneously affect the excretion of many substances, and that a spectroscopic analysis of urine can be used as a rapid and non-invasive pre-screening tool.

## Introduction

Cardiovascular disease (CVD) is the main cause of mortality worldwide, accounting for over 30% of global deaths^1^. The presence of chronic kidney disease (CKD), characterized with a decreased glomerular filtration rate (GFR), is one of the well-known risk factors for developing CVD. Recent studies, especially over the past decade, have revealed that the increased risk of CVD mortality does not only include patients with CKD, but also those having a higher-than-normal GFR, or renal hyperfiltration (RHf)^1–4^. It seems that once hemodynamic deterioration related to RHf is sustained in the glomeruli, inflammation is triggered and, eventually, sclerosis occurs^5^. Therefore, early detection of RHf is critical for favorable outcomes even though it is considered only a risk factor not a disease alone.

The detection of RHf requires the measurement of glomerular filtration rate (GFR); however, doing this is a challenging task under clinical settings. Obtaining the most accurate GFR using exogenous filtration markers (inulin, iothalamate, iohexol, etc.) in specific conditions may be considered, though not applicable to all cases^6^. Apart from this, the use of such markers is not logical in routine clinical practice due to high costs, more invasiveness, and time-consumption. Additionally, there is no commonly used acceptable GFR threshold for the diagnosis of RHf. In some studies, RHf is defined as being more than two standard deviations above the mean eGFR (estimated GFR) of normal population; whereas, others have assumed different levels of eGFR in the range between 125 ml/min/1.73 m^2^ and 175 ml/min/1.73 m^2^. Some have even stated that RHf, measured with a creatinine-based model, could not reflect its actual-state unless increased levels of filtration fraction rate (GFR/ERPF -effective renal plasma flow-) exist as well^5^. Urinary clearance of creatinine using 24-h urine samples can be used as a confirmatory analysis. However, it requires the collection 24-h urine samples and overestimates GFR at approximately 5–10 ml/min/1.73 m^2^ due to tubular secretion of creatinine^6^. Moreover, 24-h urine collection is not easy for patients and often cannot be performed accurately, especially by women and elderly patients. Taken together, it is clear that both clinicians and patients need a practical, fast, and minimally invasive method to detect the presence of RHf. Such a urinalysis method could offer new opportunities to health care providers and their patients to preserve the kidneys, especially in this new era in which drugs, such as sodium-glucose cotransporter (SGLT-2) inhibitors, are available^7^.

Owing to technological advancements in the last three decades, many substances (more than 3000) have been discovered in urine^8^ using sophisticated machines, namely mass spectroscopy (MS), MS-TOF, NMR, chromatography combined with MS (GC–MS, LC–MS/MS), and more^9–11^. Nonetheless, the clinical practice itself has not been able to keep pace with the changes as the novel techniques mentioned earlier have, the main reason being their limitations in clinical use, mainly the costs, lengthy processing, and unavailability for many laboratories. In addition, it is cumbersome to analyze every single substance for obtaining their quantitative levels in the urine. Even if analysis is carried out, interpreting the clinical meaning of these levels is a dilemma by itself. It is well known that an increase in the level of a substance can be neutralized in-vivo by the action of another substance, and the steady-state is ultimately preserved in the body. To elucidate the clinical significance of increasing or decreasing the levels of a substance, a clinician should know not only the normal levels, but also the correlations between the studied substance and others in the same environment. This is still a major task despite all the progress made in this discipline.

Attenuated total reflection-Fourier transform infrared (ATR-FTIR) spectroscopy is a widely used spectroscopic method for identifying the chemical composition of a substance. It provides molecular information about the sample structure, composition, and dynamics without the need for tags and dyes^12,13^. Lately, the technique has been used for different purposes in the analysis of biological materials, such as blood, tissue, cells, saliva, urine, etc.^12,14–19^. There are several studies in the literature using ATR-FTIR for the identification of patients for various diseases^20–25^. Infrared spectrum of urine can be a “signature” of a clinical condition due to providing a quick “snapshot” of all substances in urine and their interactions in a single “photo frame”, as opposed to quantifying each individual component therein. This feature of ATR-FTIR could provide an advantage in the case of RHf, which has the potential of changing multiple urine components rather than a single. In the present work, we investigated the urine sample properties of hyperfiltraters using ATR-FTIR spectroscopy and compared them to normofiltraters in terms of urine spectra.

## Methods

### Study population and sample collection

In this study, all samples were collected at Trakya University Hospital, Turkey. Thirty-seven Turkish individuals between 18 and 65 years of age were included in the study, which was approved by the Trakya University Human Studies Ethics Committee (TÜTF-GOBAEK 2022/24). Informed consent was obtained from each individual participating in the study. All methods and experiments were performed in accordance with relevant guidelines and regulations. Two groups, 17RHf (+) and 20 RHf (−), were set for this cross-sectional study. Those with any of the following were excluded from the study: acute infection/inflammation, diabetes mellitus, obesity, pregnancy, hypertension, urine albumin > 30 mg/day, urine protein > 300 mg/day, cardiovascular disease, peripheral artery disease, cerebrovascular disease, chronic rheumatologic disease, and thyroid dysfunction. The GFR was calculated by the data obtained from the urine samples collected for 24-h, using a standard creatinine clearance formula (Clcr) adjusted according to the body surface area^26^. The values of Clcr above140 ml/min/1.73 m^2^ [106.4 + (2 × 11.4) + 10] are defined as RHf, determined by using more than two standard deviations above the mean Clcr of the control group (106.4** ± **11.4). To increase the accuracy, 10 ml/min/1.73 m^2^ was also added because of the commonly known tubular secretion of creatinine (5–10 ml/min/1.73 m^2^) in the normal population^27,28^. The blood and spot urine samples of the cases were examined. The eGFR was calculated and recorded using the CKD-EPI (Chronic Kidney Disease Epidemiology Collaboration) formula for all the participants^29^. The participants initially collected their urine for 24-h, which was subjected to laboratory analysis. See Table 1 for the results. Next, 37 freshly collected mid-stream urine samples taken after at least 8-h fasting time on the second day morning were analyzed with a dipstick test (Mission Acon, San Diego, California). Subsequently, all samples were immediately stored at − 80 °C for ATR-FTIR analysis.

**Table 1:** Demographic, clinical, and laboratory data of the participants; RHf (−) and RHf (+) groups.

Parameters		RHf (−) Group (n = 20)		RHf ( +) Group (n = 17)		p
		Mean	SD	Mean	SD	
Gender	Female, %(n)	50% (10)		52.9% (9)		0.892*
	Male, %(n)	50% (10)		47.1% (8)		
Smoking	Yes, %(n)	30% (6)		17.6% (3)		0.299*
	No, %(n)	70% (14)		82.4% (14)		
Age, year		39.2	11.8	38.7	12.2	0.892
BMI, kg m^2^		24.3	2.9	24.9	3.0	0.594
SBP, mm Hg		109.5	6.1	118.5	10.8	**0.006**
DBP, mm Hg		70.8	6.1	75.8	10.6	0.097
MAP, mm Hg		83.6	7.4	94.5	11.4	**0.002**
PP, mm Hg		40.1	3.5	44.0	5.6	**0.014**
P, beats/min		73.4	8.4	78.6	11.4	0.131
FBG, mg/dl		90.2	7.4	87.8	6.6	0.317
Urea, mg/dl		24.1	6.5	26.3	6.0	0.294
Creatinine, mg/dl		0.78	0.17	0.68	0.20	0.135
Uric acid, mg/dl		4.5	1.13	4.5	1.1	0.881
Triglyceride, mg/dl		93.6	42.6	111.5	18.6	0.378
T. Cholesterol, mg/dl		182.9	29.3	201.3	36.4	0.097
LDL, mg/dl		119.5	20.5	130.8	25.2	0.143
HDL, mg/dl		51.6	12.4	52.8	13.8	0.798
Albumin, g/dl		4.4	0.4	4.4	0.3	0.529
Na, mmol/l		139.2	1.6	139.4	1.9	0.784
K, mmol/l		4.4	0.4	4.3	0.3	0.213
Cl, mmol/l		104.0	1.9	104.2	1.9	0.713
Ca, mg/dl		9.9	0.3	9.8	0.4	0.480
P, mg/dl		3.4	0.5	3.3	0.6	0.659
Mg, mg/dl		2.1	0.1	2.1	0.1	0.546
eGFR (CKD-EPI), ml/min/1.73 m^2^		106.1	20.1	115.11	18.5	0.136
Clcr, ml/min/1.73 m^2^		106.4	11.4	157.7	21.0	** < 0.001**
Proteinuria^δ^, mg/day		114.2	51.6	182.3	54.3	** < 0.001**
Albuminuria^δ^, mg/day Median, IQR		4.0	2.1–7.0	8.9	5.5–12.7	0.021**
Na^δ^, mmol/l		67.1	22.3	72.9	41.5	0.590

Significant values are in [bold].

Student T test, *Chi square test, **Mann Whitney U test, ^δ^24-h urine data; p < 0.05 is considered significant.

SD, standard deviation; BMI, body mass index; SBP, systolic blood pressure; DP, diastolic blood pressure; MAP, mean arterial pressure; PP, pulse pressure; P, pulse; FBG, fasting blood glucose; Clcr, creatinine clearance; eGFR, estimated glomerular filtration rate; CKD-EPI, Chronic Kidney Disease Epidemiology Collaboration Study formula.

### ATR-FTIR measurement

A Thermo Scientific Nicolet 6700 FT-IR spectrometer, equipped with a diamond attenuated total reflection (ATR, ConcentratIR2, Harrick) accessory having 10 internal reflections, and a deuterated triglycine sulfate (DTGS) detector was used for recording the infrared spectra. Before each measurement, the ATR crystal was cleaned with ethanol and double-distilled water and dried for 3 min. The background interferogram was recorded with a clean diamond surface. Five microliters of the thawed and shaken urine sample was pipetted onto the diamond and dried for 15 min using a gentle stream of N_2_ gas prior to data collection to remove excess water. All samples were measured by collecting and averaging 128 scans for a final resolution of 4/cm. A total of 128 scans with the DTGS detector takes about 2 min to complete. The dynamic changes in the water content during evaporation were visible in the spectra for the first 10 min; after that, the O–H modes did not change in amplitude. The samples were dried for 15 min to make sure that it remained stable during the measurement. The spectrometer was continuously purged with N_2_ during measurements to eliminate atmospheric variations. The absorbance spectra were recorded in the 4000–600/cm region using the spectrometer software OMNIC, version 8.2.388 (Thermo Scientific).

Each urine sample was measured in triplicate, and the average of three measurements was used for spectral analysis. The variation among different loadings of the same sample is calculated to be < 0.01 in terms of the total integrated area that accounts for pipetting errors. All data processing, analysis, and visualization were performed using OriginPro 2017 SR2 and OMNIC. Spectra were first ATR- and baseline-corrected using multi-point linear baseline at points 3999, 3400, 2400 and 1850/cm to eliminate baseline variations. Next, spectra were vector normalized to compensate for concentration differences. A second derivative calculation was performed using a 15-point Savitsky-Golay derivative with the polynomial order set to 3.

### Statistical analysis

The statistics were obtained using the IBM SPSS Statistics for Windows, Version 22.0 (IBM Corp., Armonk, NY, USA). The Kolmogorov–Smirnov and Shapiro–Wilk tests and histogram curves were used to analyze the normality of distribution. Normally distributed numerical data were reported as mean value ± standard deviation (SD), while abnormally distributed ones were reported as median value and inter quartile range (IQR). The percentages were used to express the categorical data. The differences between the two groups (normal and RHf cases) were analyzed with an independent *t*-test for numerical variables to see whether normal distribution has indeed been determined; if not, a Mann Whitney U test was used. Furthermore, the correlations between the numerical variables were analyzed with Pearson and/or Spearman tests. A Chi-Square test was used in the comparison of the categorical variables. Statistical significance was considered when the p value was detected as < 0.05.

### Principal component analysis

Principal component analysis (PCA) is an *unsupervised* technique used to process and reduce the dimensionality of high-dimensional datasets^30^. It can detect any relationships without presumption on the priority among the multiple data. High- and low-frequency regions of the pre-processed spectra were used for PCA separately. Additionally, the one-way Analysis of Variance (ANOVA) followed by Fisher’s Least Difference Significant (LDS) post hoc test was used to find the significance of difference for the selected spectral markers. Statistical significance was considered when the p value was detected as < 0.05.

## Results and discussion

The means of Clcr in the RHf (+) and (−) groups are 157.7 ± 21 and 106.4 ± 11.4 ml/min/1.73 m^2^, respectively, and significantly different (p < 0.001), while the eGFR (CKD-EPI) of both groups is similar (115.11 ± 18.5 and 106.1 ± 20.1 ml/min/1.73 m^2^, respectively). The levels of proteinuria (182.3 ± 54.3 and 114.2 ± 51.6 mg/day) and albuminuria (*Median, IQR*; 8.8, 5.5–12.7 and 4.0, 2.1–7 mg/day) in the 24-h urine samples are also significantly different (p < 0.001 and 0.018, respectively). In the clinical data, the systolic blood pressure (SBP) (RHf (+) group, 118.5 ± 10.8 mm Hg; RHf (–) group, 109.5 ± 6.1 mm Hg), mean arterial pressure (MAP) (94.5 ± 11.4; 83.6 ± 7.4 mm Hg), and pulse pressure (PP) (44.0 ± 5.6; 40.1 ± 3.5 mm Hg) are significantly different; while the diastolic blood pressure is statistically similar (p = 0.006, 0.002, 0.014 and 0.097, respectively). The correlation analysis between Clcr and SBP, MAP, and PP showed that they are positively correlated with Clcr (*r* = 0.453, p = 0.005; *r* = 0.484, p = 0.002 and *r* = 0.358, p = 0.03, respectively). Proteinuria also has a positive correlation with Clcr (*r* = 0.387, p = 0.018), while albuminuria does not have any such relationship. The two groups are similar in terms of gender, age, BMI, smoking, and other laboratory parameters. The demographic, clinical, and laboratory data are summarized in Table 1.

**Figure 1:**
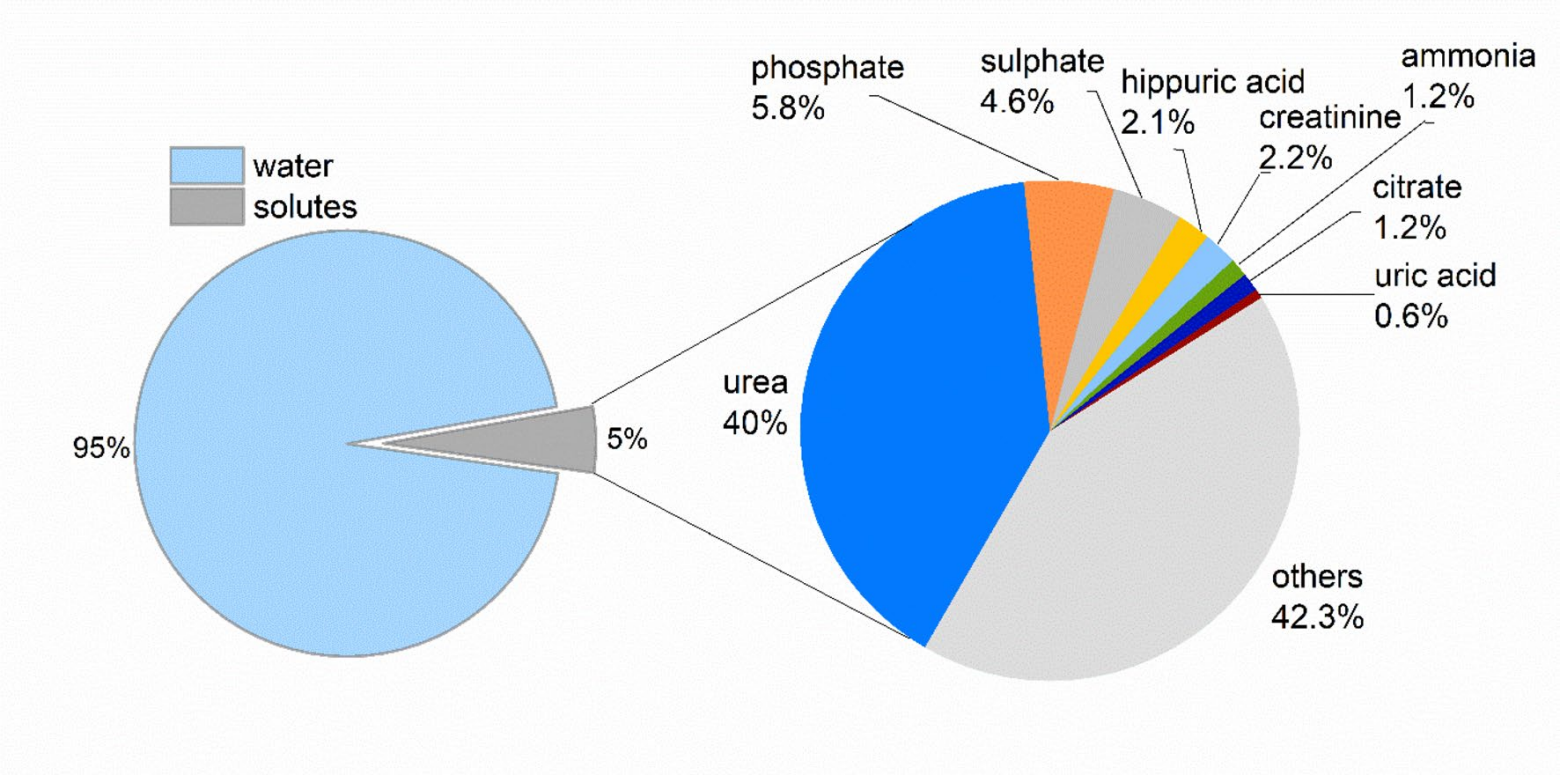
The composition of human urine. The percentages reflect the mass ratio of each urine component with respect to the total mass of 1.5 L-urine. The others (42.3%) represent hundreds of different molecules, each contributing less than 0.5% to the total urine composition in the same chart^32–34^.

In addition to the 24-h urine samples and their laboratory analyses, spot urine samples collected from the same participants on the second day morning were analyzed with ATR-FTIR spectroscopy. Using this technique has the advantage of showing all the molecular groups in one frame, but it also has the disadvantage of not providing information on ions^31^. Among the components of urine, urea is the most dominant one in the infrared spectrum due to its strongly absorbing functional groups, as well as its abundance relative to other components (Fig. 1).

The other urine components that are directly visible without mathematical enhancements are uric acid, creatinine, citrate, ammonia, phosphate, and sulphate groups. Although lipids are not expected in normal urine samples, their existence can still be directly observed^35,36^. On the other hand, pathological amounts of glucose or protein meaningful in clinical practice cannot be observed directly unless very high; this is because the characterizing peaks overlap with those of other components in the urine. To detect glucose, protein, and other similar urine components, various spectral enhancement methods have been successfully used in the literature^37–39^. For this study, those with abnormal glucose level and proteinuria above 300 mg/day in their urine were excluded altogether.

**Figure 2:**
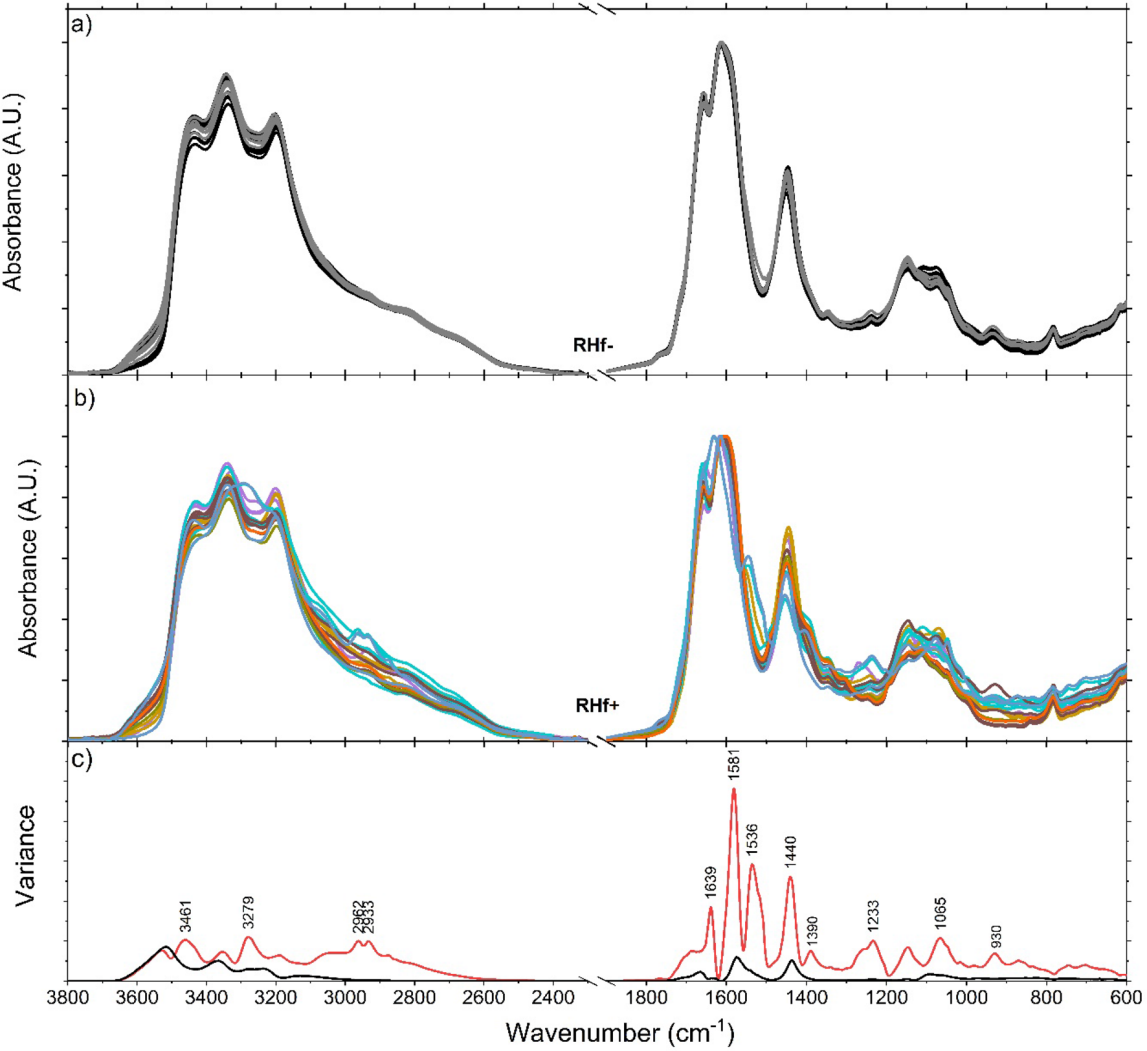
Overlaid urine spectra of RHf (−) (n = 20) and RHf (+) (n = 17) participants. Each spectrum is the average of triplet measurement of the same sample. Spectra are ATR- and baseline-corrected and normalized to the 1610/cm urea peak. Raw spectra are also provided in Fig. S1 (Supporting Information). Lower panel (**c**) shows the calculated variances of the control group (black) and the RHf (+) spectra (red).

A full spectrum analysis of spot urine samples was carried out to distinguish the RHf (+) and (−) groups using the urine components directly observable from the absorbance spectra. Figure 2-top panel (Fig. 2a) shows the spectra of the control group. As can be seen, they all show similar properties and the same principal absorption bands. Middle panel (Fig. 2b) demonstrates the spectra of participants diagnosed with RHf. It can be observed that the spectral properties of each individual differ from others as well as from those in the control group. This spectral diversity among RHf (+) individuals reflects the compositional diversity of their urines and there is no observable linear relationship between peak profiles and individuals’ Clcr levels. Comparison of variances shows that the spectra of RHf (+) group deviate from the control group at multiple points (Fig. 2c).

PCA is applied to the pre-processed IR spectra for two separate regions as high-frequency and low-frequency, defined as 3800–2500/cm (Fig. S2-Supporting Information) and 1800–700/cm (Fig. 2), respectively. The control group samples are tightly clustered whereas the RHf (+) samples exhibit a wider distribution relative to the control samples. PC1, PC2 and PC3 explain 77.7% of total variance. The scores of RHf (−) samples lie on the positive side of the PC2 scores, while those of RHf (+) are mostly on the negative side. It is important to note that PCA classified the control group as close to one-another despite spectral variations due to age, gender, and dietary habits. It is also evident that the in-class variation of the RHf (+) samples is higher than that of the RHf (−) samples.

**Figure 3:**
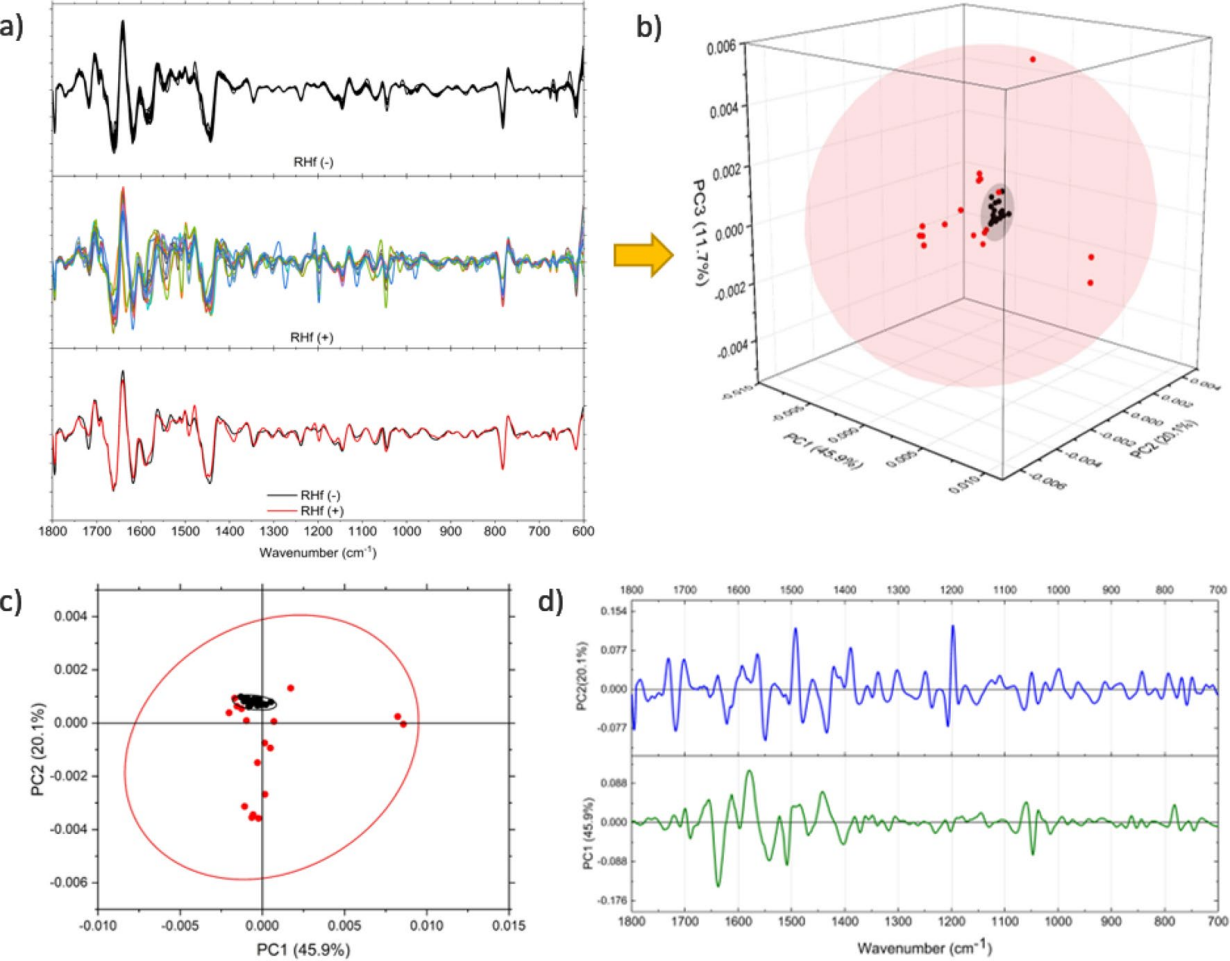
The second derivative of spectra (**a**) were used for PCA scores plot for the 1800–700/cm low-frequency region. The average of second derivative profiles for each group is given in the bottom panel. 3D scatter plot (**b**) and bipolat (score (**c**)-loading (**d**)) display the clustering of the control group samples on the negative PC2 axis. Positive peaks in the PC2 loading plot show the spectral positions that contributed to the displayed classification.

**Figure 4:**
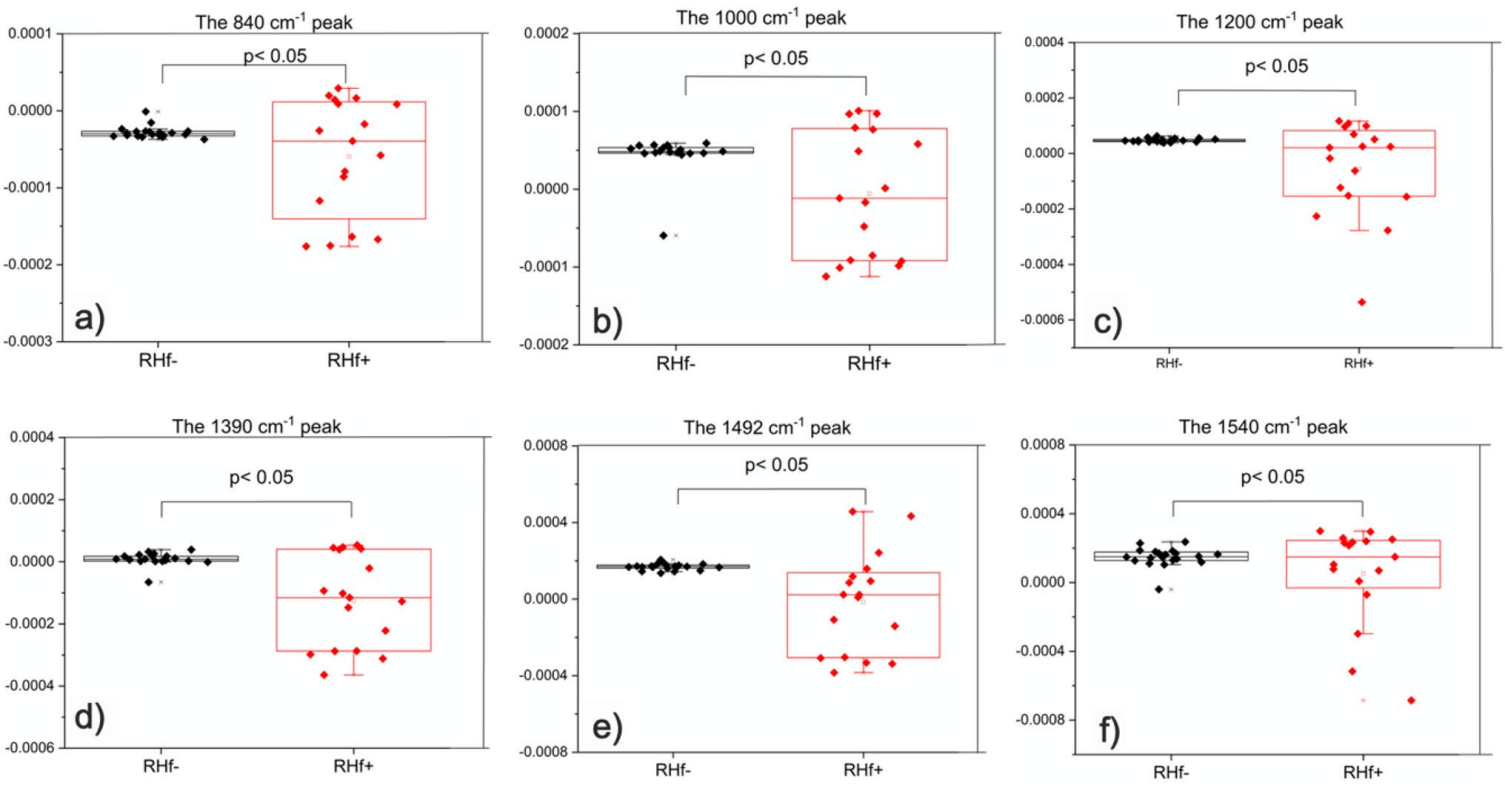
Intensity changes of peaks selected from PCA loading plot. All peaks are significantly different compared to the control group. Second derivative of spectra were used for calculating the mean** ± **SD represented by the boxes. Data was analyzed by one-way ANOVA and Fisher’s post hoc test.

In the clinical understanding of hyperfiltraters, there is heterogeneity among these individuals in terms of the cause and the results. Urine composition shows this heterogeneity as well. Evaluation of Figs. 2c and 3a (second derivative of spectra), Fig. 3b (clustering of normofiltraters is clearly observed on 3D scatter plot of PCA scores), Fig. 3c (clustering is seen especially based on PC2 axis), Fig. 3d (loading plot of PCA) reveals that there are common differences between the spectra of RHf (+) and (−) groups. Table S1 (Supporting Information) lists these peaks and their proposed functional group assignments. The vibrations at ~ 840, 1000, 1200, 1390, 1492, and 1540/cm contributed mostly to the spectral separation of the two groups along the PC2 axis (Fig. 3c). These peaks mostly originate from urea, uric acid, creatinine, citrate, sulphate, and phosphate groups in a healthy population. On other hand, contribution of proteins at 1545/cm and purines and/or purine derivatives at 1240/cm and 1100–970/cm should also be taken into consideration in our RHf (+) population. The statistical significances of all six peaks are further tested by one-way ANOVA, followed by Fisher’s LDS post hoc test (Fig. 4), and all are found to be statistically significant in discriminating RHf (+) samples.

### Origin and contribution of the major IR peaks to the observed spectral diversity

When the spectral variances are compared, the degree of changes at any given spectral position is much higher with the RHf (+) group (Fig. 2c). This observation implies that RHf is not characterized by a single biomarker band but, instead, it is an observable spectral deviation from the normal (control group) at multiple points. In order to study the sources of deviations, the raw spectra (Fig. S1-Supporting Information) are analyzed by selecting peaks so that each one represents a different urine component, and that each is relatively more isolated from the absorptions of other urine components. In this respect, five peaks are selected and used as presented in Table 2. The rationale for using raw spectra rather than pre-processed ones for peak analysis is to show that the assessment of urine components as such provides a practical method that can be implemented in the clinics. However, the proposed method uses the advantage of using 8-h-fasting morning urine samples due to their comparable solute concentrations, but its applicability to spot urine samples collected during the daytime is yet to be tested. The changes at the selected peak positions are observable from the absorbance spectra. Each peak is studied by measuring either the peak-height or peak-area from the absorbance spectra. A proper baseline is selected for each to eliminate concentration differences and baseline variations. At these positions, the RHf (+) spectra are significantly different than the RHf (−) ones, as shown by the Mann Whitney U test (Table 2).

**Table 2:** Urine peak analysis summary.

Peak position (/cm)	Method	Baseline	Control average ± SD (n = 20)	RHf (+) average ± SD (n = 17)	# of samples out-of-range	p value
783/1450	Integrate btw. 765 and 788	Line btw. 765 and 811	0.469 ± 0.020	0.504 ± 0.067	12 (70%)	< 0.001
	1447 and 1470	Line at 1470	Min–max: 0.442–0.508	Min–max: 0.379–0.660		
933	Integrate btw. 936 and 924	Line btw. 905 and 961	0.845 ± 0.135	0.470 ± 0.516	15 (88%)	< 0.001
			Min–max: 0.570–1.246	Min–max: 0–1.533		
1346	Integrate btw. 1346 and 1327	Line btw. 1327 and 1361	0.323 ± 0.020	0.380 ± 0.155	15 (88%)	< 0.001
			Min–max: 0.277–0.372	Min–max: 0–0.708		
1390	Height at 1390	Line at 1360	0.134 ± 0.017	0.178 ± 0.034	8 (47%)	< 0.001
			Min–max: 0.095–0.162	Min–max: 0.134–0.242		
1621	Height at 1621	Line at 1750	1.843 ± 0.066	1.755 ± 0.199	7 (41%)	0.009
			Min–max: 1.675–1.931	Min–max: 1.359–2.002		

The number of out-of-range samples is determined based on the normal range defined by the control group min–max (limit) values. Statistical analysis was carried out using Mann Whitney U test.

#### The 783/cm peak

It is mainly originating from uric acid, but urea also contributes to the overall shape. Therefore, the limits of integration are chosen so as to include contributions more from the uric acid and less from the urea. As a second step to eliminate the changes due to urea content, the area of the 783-peak is divided by that of the 1450-peak, which is mostly due to urea. Among the RHf (+) individuals, ten people exhibit an increase in the 783-peak area compared to the control group in this study. The decreased area of the same peak is detected in two other spectra in addition to the ten spectra of the RHf (+) cases. However, the other uric acid peaks do not show such a decrease; thus, the cause concerning this decrease in these two spectra from the RHf (+) group is yet to be found. The decrease in the 783-peak could as well be due to the lack of a superposing band of another urine component. In total, 70% of our cohort shows changes in the 783-peak absorbance, mostly by an increment.

As the main source of 783-peak, uric acid (C_5_H_4_N_4_O_3_) is formed as a result of purine catabolism^40^; it is synthesized mainly in the liver, intestine, and endothelia. The sources of purine are either exogenous (food) or endogenous (damaged or dead cells)^41^. While the kidneys excrete 70% of the daily uric acid (UA) load, 30% is catabolized by the intestines. It has been shown that hyperfiltration in the early stages of diabetic nephropathy may contribute to UA excretion in diabetic patients^42^. On the other hand, some data show that both UA and sodium excretion may decrease in cases with hyperinsulinemia and, particularly, with metabolic syndrome^43^. In addition, the presence of tubular damage caused by any reason may increase the UA excretion, although there is no sign of renal injury in any of the participants^44^. Another important factor that affects UA excretion is purines taken with daily diet. Animal-based protein consumption may contribute to increased excretion of UA^45^. When the control group and the RHf (+) participants were compared in our study, the BMI, serum lipids, and serum UA levels were statistically similar.

#### The 933/cm peak

Itmainly represents the phosphate groups. The area of this peak is compared between the control and the RHf (+) group. Accordingly, the spectra of four RHf (+) participants show signs of slightly increased 933-peak area; however, those of 11 participants show decreased peak areas with respect to the control group. In our cohort of 17 RHf (+) cases, 15 individuals (%88) show variations, mostly a decrease, at 933-peak.

Phosphate (P_i_) plays very important roles in the production and transmission of energy, intracellular homeostasis, and healthy growth of skeleton. Therefore, both the blood P_i_ level and urinary P_i_ excretion are under strict control within the body. Urine P_i_ excretion is approximately 1 g/day with a typical western-type diet^46^. A variety of factors can affect the tubular re-absorption of P_i_ such as dietary intake, parathyroid hormone, vitamin D, FGF23/Klotho, other hormones (insulin, growth hormone, IGF-1, glucocorticoids, ANP, prostaglandins etc.), and non-hormone mechanisms such as fasting, plasma calcium levels, acid–base balance, volume status, pharmaceutical drugs, etc. In a study from Korea, 33,210 individuals were evaluated, and it was revealed that participants with severe vitamin D deficiency (defined 25-OH vitamin levels < 10 ng/ml) had higher rates of RHf than those with adequate vitamin D in their serum (5.8% and 5%, respectively; p < 0.001)^47^. Many studies, but not all, have concluded that vitamin D deficiency may increase the rate of progression of renal injury^48^. Vitamin D deficiency is quite common in adult populations as well; in a meta-analysis involving 111,582 subjects, a total of 40 studies were evaluated in Turkey, and it was shown that the frequency of vitamin D deficiency (defined as < 30 ng/ml in plasma) is 63.5% in adults^49^. FGF23, together with Klotho, inhibits the 1-OH-lase that is crucial for the activation of vitamin D on the one hand, and contributes to keeping the serum P_i_ level within a narrow range by increasing the excretion of P_i_ on the other hand. Decreased levels or dysfunction of FGF23 and/or Klotho is associated with decreased urinary P_i_ excretion^46^. In the present study, not all the parameters that could affect the urinary P_i_ levels were evaluated. Nonetheless, one or more of the afore-mentioned mechanisms might be involved in RHf and/or changes in the urinary P_i_ level.

#### The 1346/cm peak

Creatinine is another component of urine that can be easily followed with infrared spectroscopy. Although there are many peaks originating from different vibrational modes in the structure of creatinine, the peaks at 1490, 1346, 1242, and 842/cm are relatively more isolated from contribution by other urine components. For this reason, the 1346-peak is chosen to better understand the creatinine-induced changes within the spectra. Calculating and comparing the area of this peak revealed that eleven spectra show an increase in the peak area, and four show decreased areas with respect to the control group. Out of 17 RHf (+) individuals, 15 (88% of the cohort) spectra show changes in the 1346-peak area with respect to the control group.

Creatinine (C_4_H_7_N_3_O) is produced mainly from creatine in muscles^50^. Therefore, quantifying the amount of creatinine in 24-h urine is an accepted method for determining body muscle mass^51^. Creatinine is excreted without being reabsorbed by the tubules after it is filtered from the glomeruli, and a small amount of creatinine (8–10% in the presence of normal GFR) is excreted via tubular secretion^28^. In other words, the main determinant of the creatinine level (90%) in the urine is the muscle mass of the body; the remaining part (10%) comes from daily protein consumption in the diet^28,52^. It has been shown that high-protein diet has a linear positive relationship with the excretion of not only creatinine, but also urea, which is a breakdown product of proteins. Every 5 g of urea is accompanied by an increase in the creatinine clearance of 30 ml/min/1.73 m^2^^52^. Thus, both RHf and increased creatinine excretion seem to be partially related to the dietary protein intake.

#### The 1390/cm peak

It is mainly due to citrate, but it overlaps with strong urea absorption at ~ 1450/cm. Although an increase at the 1390-peak absorption can be easily observed in the spectrum, detecting a decrease is more challenging. The other characterizing peaks of citrate can be listed as 1573, 1300, and 1256/cm. The 1573/cm peak appears under the large urea peak and, thus, is obscured. The 1390-peak is relatively easier to work with compared to the other peaks of citrate. The amplitude changes at this position can be used to observe citrate variations. In our cohort, the spectra of eight individuals showed increased 1390-peak absorbance relative to the control group. The rest of the RHf (+) group did not show a significant alteration. The acid–base status of the body directly influences the urinary citrate levels. More specifically, the acidosis in tubular cells in kidney, which is the consequence of systemic acidosis, decreases the urinary citrate levels. Therefore, higher urine pH, meaning lower systemic acid loads, is related with higher urinary citrate^53^. In addition, there are many factors such as high protein diet, diarrhea, malabsorption, exercise, starvation, potassium depletion, transport competitors (malate, fumarate etc.), metabolic inhibitors (fluorocitrate, maonate, etc.), medications (lithium, acetazolamid, amiloride, vitamin D, calcium, angiotensin-converting enzyme inhibitors, etc.), and genetic factors that lead to the alteration of urinary citrate levels^53^. All (n= 37) participants in this study were healthy medically; this implies that diet habits (protein and acidic beverage intake) and genetic features might be responsible for the variation in this region in our subjects.

#### The 1621/cm peak

Urea absorption dominates the spectrum of the urine samples. Both in the high-frequency and in the low-frequency regions of the spectrum, the urea content can be followed from the absorbance changes at positions 3430, 3256, 3336, 1675, 1621, 1594, 1463, and 1146/cm. The absorbance at 1621/cm is used to determine the urea variations in the RHf (+) group. While three spectra showed increased absorbance, four showed decreased absorbance relative to the control group. In total, 41% of the cohort shows changes at 1621/cm (Table 2). The remaining 10 individuals in the RHf (+) group did not show variations out of the range defined by the control group.

Urea (CH_4_N_2_O) is the most abundant substance in the urine and is the main nitrogen-breakdown product of the organism^54^. Its excretion mainly depends on GFR and the intratubular ultrafiltrate flow rate. Urea is not secreted at any site within the tubular system, but it may be reabsorbed by tubular re-absorption, especially if the ultrafiltrate flow is reduced. In general, when the urine formation rate is above 2 ml/min, 60% of urea is excreted in the urine, while this rate decreases to 20% when < 0.5 ml/min^54,55^. As a result, excessive excretion of urea is an expected situation, especially in RHf (+) subjects. Besides, some conditions may affect both urea excretion and RHf at the same time. For instance, the protein intake within the diet increases both GFR and the urea formation/excretion according to the data in the literature^1,56^. In addition, it has been revealed that vasopressin levels, which increase water absorption in the collecting tubules, affect blood and urine urea levels^54,55^. The urea excretion of hyperfiltraters—three have increased and four have decreased excretion—in our study is diverse, which can be attributed to variable protein and water consumption.

### Other spectral observations

The analysis of each individual spectrum revealed unique differences in some of the RHf ( +) group members. For example, one spectrum showed a clear peak at 1541/cm in the absorbance spectrum. Analysis of the same peak from second derivative forms showed that the RHf (+) group has a higher standard deviation (Fig. 4f). Calculated variances, shown in Fig. 2c also confirms the variation of this peak,which has been assigned to the protein amide II mode in a previous study^16^. Laboratory analyses of these particular individuals also show increased total protein in 24-h-urine (> 200 mg/day), which agrees with the spectroscopy findings. The relationship between RHf and urinary albumin and protein excretion has been demonstrated in the literature^57,58^. Although the albumin excretion of all participants was in normal range (< 30 mg/day) in our study, it is observed that there is a statistically significant difference between those with or without RHf. The situation is also similar for proteinuria; a study conducted among native Austrians revealed that normoalbuminuric hyperfiltrater (mGFR ≥ 125 ml/min/1.73 m^2^) individuals significantly develop overt albuminuria when compared to normofiltraters at the end of 3 years of follow-up (60% and 32%, respectively)^59^. It has been shown that RHf alone may be an indicator of renal damage in individuals who are pre-hypertensive and pre-diabetic^60^. In the present study, it is shown that despite not finding a difference in FBG and BMI, the SBP values were higher in participants with RHf than in those without. Moreover, this significant statistical difference was similar for the MAP and PP. The most powerful correlation was found between MAP and Clcr followed by SBP and PP. In clinical practice, the accepted general threshold for the diagnosis of hypertension is ≥ 140/90 mm Hg, although a lower cut-off value has been recommended by some authors. The main reason for this recommendation is the observations of increased cardiovascular mortality at lower blood pressure levels. In fact, a study showed that the risk begins to increase from the SBP level as low as 90 mm Hg^61^. Interestingly, this increased mortality was demonstrated even in the absence of traditional risk factors^62^. Our findings about the hemodynamic parameters imply that RHf might be associated with increased cardiovascular mortality.

In another spectrum from the RHf (+) group, there are four peaks at positions 1270, 1113, ~ 1040, and 980/cm that are unique in terms of the peak profile and magnitude. They clearly stand out in the absorbance spectrum, but their origin cannot be explained with the afore-mentioned urine components. In a previous study, the 1278/cm peak is assigned to the N–H deformation in purines^63^; though, it can also be assigned to the P–O or P–CH_3_ vibrations^64^. The peaks at 1113/cm can be tentatively assigned to the phosphate groups having slightly different electro negativity on the phosphate^64^. The peaks at 1040 and 980/cm can be assigned to C–O and C–C stretching modes in purines.

**Figure 5:**
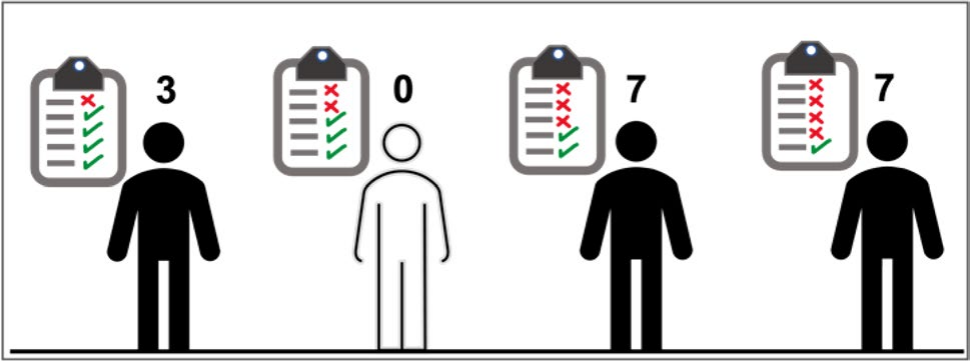
The spectral changes for five urine components (urea, citrate, creatinine, phosphate groups, and uric acid) are counted for each participant. Seven individuals showed a change in three of their urine components and another seven showed a change in their four different urine components.

In most of the participants, there is a variation at positions 933 and 1346/cm, pointing out to changes in the phosphate groups and creatinine, respectively, as regards the normal range (Table 2). When the findings presented in Table 2 are reconstructed to see the list of changes in five selected peaks for each participant, it is found that the likelihood of having three or four urine components changing simultaneously (going out of the normal range) is higher than having a change in a single urine component (Fig. 5). Therefore, RHf is mostly characterized by producing changes in multiple urine components. In this respect, FTIR spectroscopy can be used as a fast-screening method to compare the spectrum of a person against a collection of control spectra to observe these changes.

## Conclusion

The trend observed in the studies in the literature regarding a medical condition is mostly an effort to detect a single biomarker using sophisticated devices. However, an increased or decreased substance (marker candidate) may not always be associated with clinical abnormality or diseases alone, and sometimes looking at the whole composition may provide additional data. This approach comes to the fore especially in cases such as RHf, which is not yet considered a disease by itself and can not only be affected by many conditions, it can also influence the excretion of many substances in the urine.

This study shows that the urine composition of RHf (+) individuals is different than RHf (−) ones. Thus, FTIR spectroscopy could serve as an effective tool for identifying people with RHf. In PCA scatter plot, normofiltraters are tightly clustered, as opposed to highly scattered hyperfiltraters that conveniently reflects the spectral diversity among these individuals. Both the second derivative and absorbance spectra were used to explain the diversity of RHf (+) group and their difference from the control group. Using the second derivative spectra, the differences at positions 1540, 1492, 1390, 1200, 1000, and 840/cm between the two groups were shown to be statistically significant. Analysis of the absorbance spectra showed that RHf can be characterized by changes in the natural urine components that are out of the normal range defined by the control group. In this respect, five different peaks, each representing a different urine component, are selected for further analysis. Among the hyperfiltraters, 88% of the population had differences at 933/cm, 88% at 1346/cm, 70% at 783/cm, 47% at 1390/cm. These positions were tentatively attributed to phosphate groups, creatinine, uric acid, and citrate, respectively.

There are some limitations to this study. The first is the small sample size, purposely kept this way due to the study being a preliminary one. In light of the information obtained, another study with a larger group can be designed. The second limitation of the study is the use of creatinine clearance as the method for GFR determination. Although this method is more practical, it does not yield results as conclusively and accurately as the gold-standard inulin and/or scintigraphy analysis. Nevertheless, this weakness was tried to be reduced by setting the hyperfiltration threshold to a value as high as 140 ml/min/1.73 m^2^ by taking into account the tubular secretion. Lastly, the dietary characteristics of the participants were not recorded. All individuals were evaluated clinically and confirmed to be healthy, but if their dietary habits and water consumption had been known, a more accurate interpretation of data could have been made. As a result, future studies will be designed accordingly.

Out of the 17 RHf (+) individuals participated in this study, 13 of them showed obvious spectral differences even from the absorbance spectra without pre-processing. With this motivation, the method proposed in this paper is simply to compare urine absorbance spectrum of an individual against a collection of a control group. ATR-FTIR provides valuable data in a very short amount of time (only a few minutes) with a single drop of urine. This way, it can serve as a rapid and non-invasive pre-screening tool that aids the clinician. Nowadays, evidence is growing regarding SGLT-2 inhibitors that have the ability to restore GFR to normal levels partly or completely in the kidneys. Taken together, the early diagnosis of RHf using a widely available and practical method in clinical settings would offer a chance to healthcare providers and clinicians for timely intervention in RHf (+) cases and normalizing the GFR level. However, further well-designed studies are required to establish the usefulness of FTIR in urine analysis as a promising method for future applications.

## Supplementary Information

Supplementary Information.

## Data Availability

The datasets used during the current study available from the corresponding author on reasonable request.
