# Supplementary material for: Urinalysis of individuals with renal hyperfiltration using ATR-FTIR spectroscopy
Source: Sci Rep. 2022 Dec 3;12:20887. doi: 10.1038/s41598-022-25535-1 (PMC9719484; doi:10.1038/s41598-022-25535-1)
Supplement: Supplementary file 1 — Supplementary Information. [file 41598_2022_25535_MOESM1_ESM.docx]

**Urinalysis of Individuals with Renal Hyperfiltration Using ATR-FTIR Spectroscopy**

İlhan Kurultak^1^^[[1]](#footnote-2)^*, Neslihan Sarigul^2^, Nil Su Kodal^3^, Filiz Korkmaz^4^

^1^Department of Nephrology, Faculty of Medicine, Trakya University, Edirne, Turkey

^2^Institute of Nuclear Science, Hacettepe University, Ankara, Turkey

^3^Department of Internal Medicine, Faculty of Medicine, Trakya University, Edirne, Turkey

^4^Biophysics Laboratory, Faculty of Engineering, Atilim University, Ankara, Turkey

Raw spectra collected in this study is shown in Figure S2. Each spectrum is the average of triplicate measurement of the same sample.


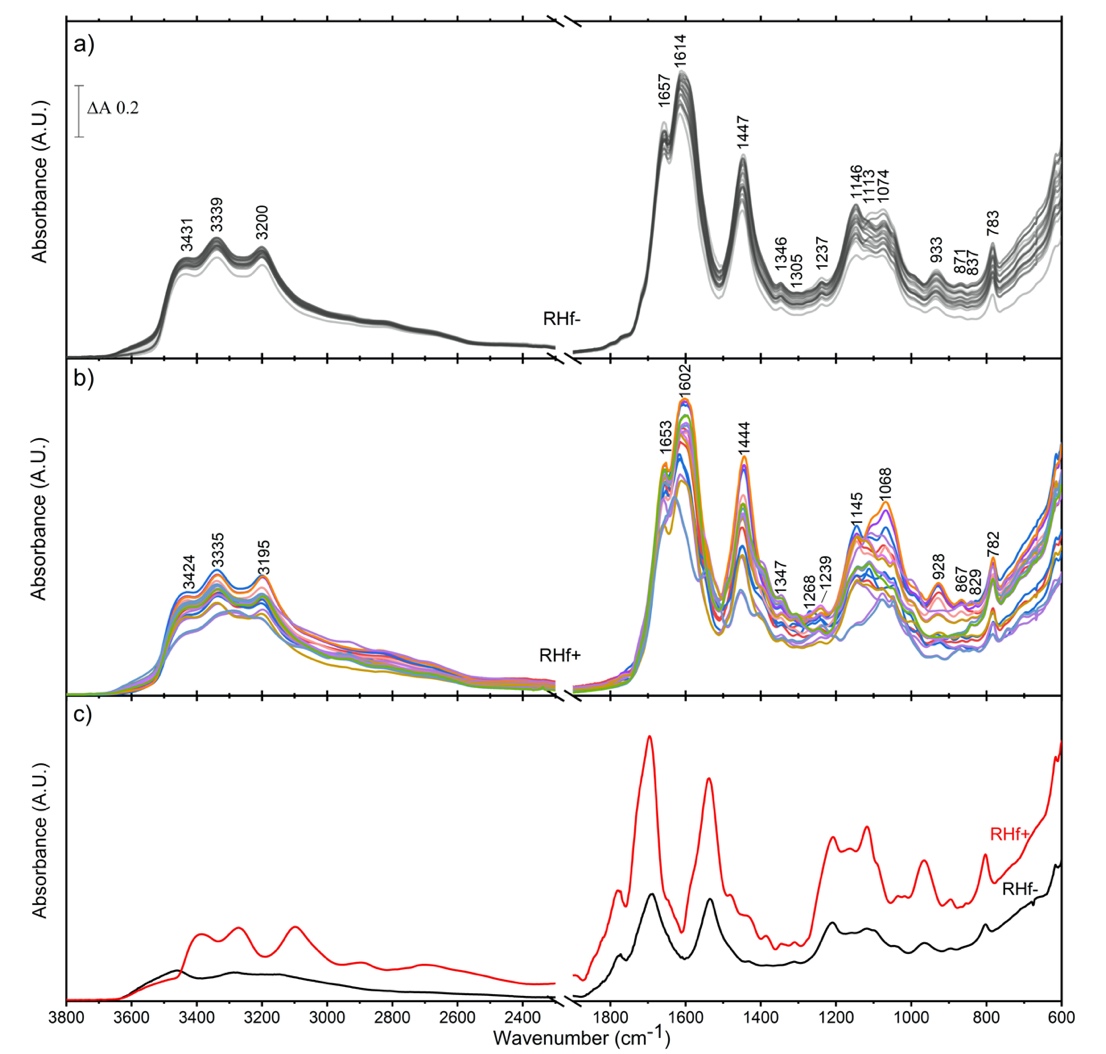


**Figure S1.** Raw infrared spectra collected for the RHf (-) group (top panel) and the RHf (+) group (middle panel). Lower panel compares the calculated spectral variance of each group.

**Principle Component Analysis of the High-Frequency Region:** The second derivative of spectra (Fig. S2a) are used for PCA. The scores scatter plot of the high-frequency region (3800-2500 cm^-1^) indicates that RHf (-) is grouped on the positive axis of PC1, which accounts for 50.2% of the total variance. Although the RHf (+) is located on the opposite side of the same axis, the distance between the two groups is close. Additionally, there are RHf (+) samples classified within the confidence ellipse of the RHf (-) samples. These samples do indeed have less degree of spectral difference with respect to those in the same group. According to the previously published data, urine components show broad peaks in the high frequency region^1^, which makes this region not ideal for quantitative analysis. Therefore, the difference between the control group and the RHf (+) group cannot be assigned to a specific urine component; rather, it is a cumulative effect of many components.


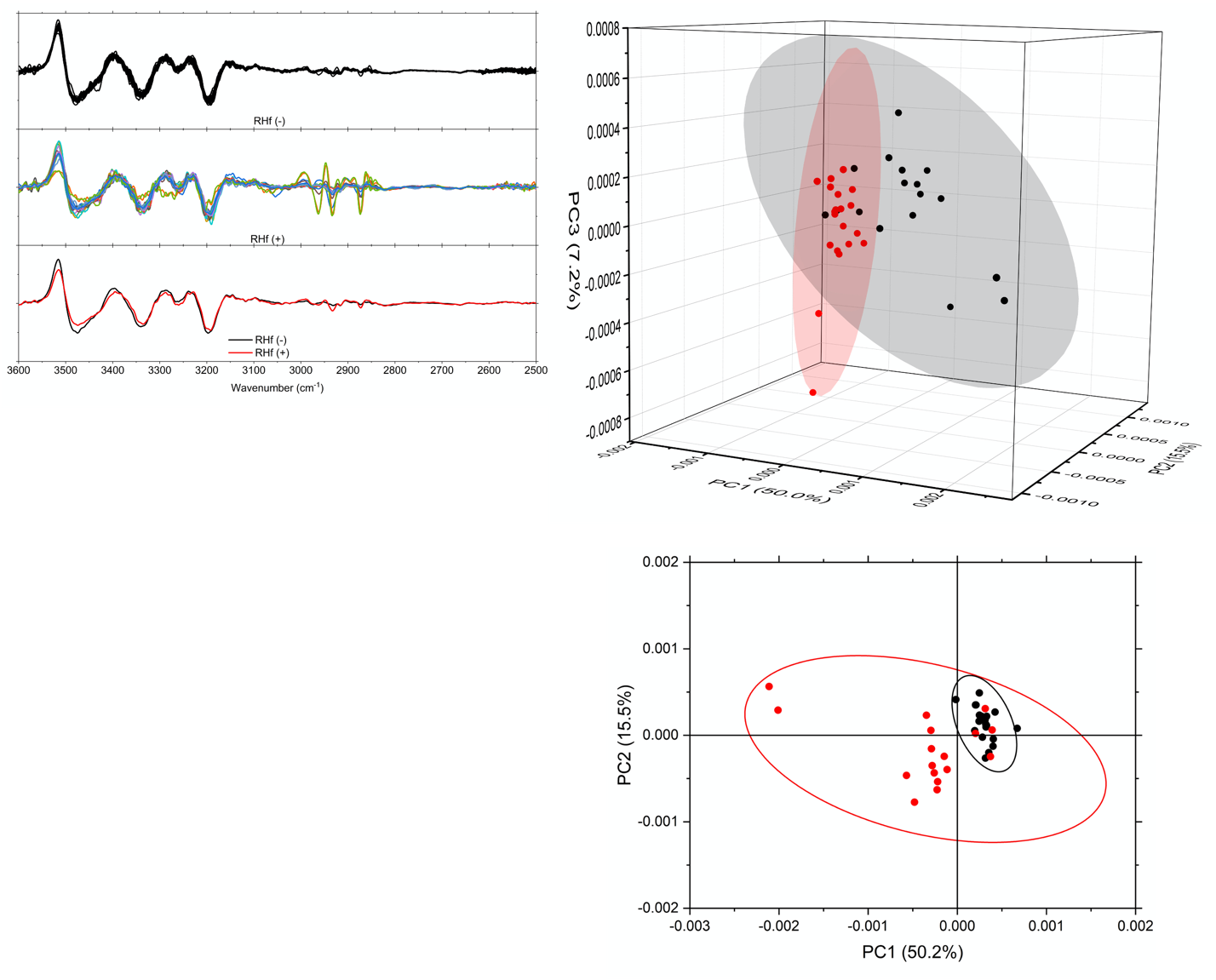


**Figure S2.**Second derivative forms of spectra from both groups are shown in the top and middle panel (a). Bottom panel compares the average of the two groups. Three-dimensional (b) and bipolat (c) PCA scores plot for the high-frequency (3800-2500 cm^-1^) region. First three principal components explained 72.9% of total variance.

**Table S1**. Band positions and their tentative assignments shown in PCA loading plots for the low frequency region.

| Wavenumber (cm^-1^) | Assignment^2,3^ | Possible origin^4,1^ |
| --- | --- | --- |
| 782 | N-H wagging, C-H (ring) bending | Urea, uric acid, with baseline contributions from phosphate and sulphate groups |
| 938 | P-OH bending | Phosphate groups (centered at 933 cm^-1^) |
| 970 | C-C stretching |  |
| 992/1010 | S-O stretching, C-H wagging, C-N stretching | Sulphate groups and urea with baseline contribution from phosphate groups |
| 1050 | C-O stretching |  |
| 1065 | P-Oand S-O stretching | Phosphate and sulphate groups withbaseline contributions from urea. |
| 1080 | PO_2_ symmetric stretching |  |
| 1145-1157 | C-NH_2_, C-O, S-O stretching, NH_2_ rocking | Urea with minor contribution from uric acid and citrate with baseline contributions from phosphate and sulphate groups |
| 1240 | PO_2_asymmetric stretching |  |
| 1309 | (O=C)-O | Oxalate with contributions from citrate and uric acid (at 1300 cm^-1^) |
| 1351 | C-H bending (ring), C-N stretching | Uric acid (centered at 1346 cm^-1^) |
| 1390 | C-OH stretching | Citrate with contribution from NH_4_ deformation mode of ammonium at 1400 cm^-1^ |
| 1441/1446 | C-H bending, C-O stretching, C-N stretching | Urea (centered at 1463 cm^-1^), uric acid and creatinine |
| 1545 | C-N stretching, N-H bending | Protein amide II band |
| 1582/1588 | C-C stretching, N-Hbending | Uric acid, citrate and creatinine with contribution from 1594-urea band |
| 1608 | N-H deformation, C-N-H vibrations | Urea, creatinine and citrate |
| 1652/1663 | C=O stretching, C=N stretching, NH_3_^+^ deformation | Urea (NH_2_ bending mode centered at 1676 cm^-1^), uric acid, creatinine, ammonium and proteins with baseline contribution from citrate |
| 1715 | C=O stretching | Uric acid, ammonia, lipids, ketones |

**References**

1. Sarigul N, Korkmaz F, Kurultak İ. A New Artificial Urine Protocol to Better Imitate Human Urine. *Scientific Reports*. 2019. doi:10.1038/s41598-019-56693-4

2. Colthub N, Daly L, Wiberley S. *Introduction to Infrared and Raman Spectroscopy*. Third Edit. Academıc Press, Inc.; 1990.

3. Grdadolnik J, Maréchal Y. Urea and urea–water solutions—an infrared study. *Journal of Molecular Structure*. 2002;615(1-3):177-189. doi:10.1016/S0022-2860(02)00214-4

4. Sarigul N, Kurultak İ, Uslu Gökceoğlu A, Korkmaz F. Urine analysis using FTIR spectroscopy: A study on healthy adults and children. *Journal of Biophotonics*. 2021. doi:10.1002/jbio.202100009

1. *Correspondence. E-mail: ilhankurultak@yahoo.co.uk [↑](#footnote-ref-2)
